# Role of Hemoperfusion With HA330 in the Management of Severe Dengue Shock Syndrome

**DOI:** 10.1155/jotm/9928338

**Published:** 2026-01-05

**Authors:** Tananat Virojtriratana, Kanruetai Na Nan, Rujipat Samransamruajkit

**Affiliations:** ^1^ Division of Critical Care Medicine, Department of Pediatrics, Faculty of Medicine, Chiang Mai University, Chiang Mai, Thailand, cmu.ac.th; ^2^ Division of Critical Care Medicine, Department of Pediatrics, Faculty of Medicine, Chulalongkorn University, Bangkok, Thailand, chula.ac.th

**Keywords:** dengue shock syndrome, hemoperfusion, hemophagocytic syndrome, multiple organ dysfunction syndrome

## Abstract

**Introduction:**

Infection‐associated hemophagocytic syndrome (IAHS) is a rare complication in dengue infection that leads to multiple organ dysfunction (MOD) and increased mortality rates. The early use of hemoperfusion with hemoadsorption and standard treatment may reduce inflammation and prevent mortality. This study investigates the therapeutic effectiveness of a combination of hemoperfusion with hemoadsorption and multimodal therapy, including intravenous immunoglobulin (IVIG), corticosteroids, therapeutic plasma exchange (TPE), and/or extracorporeal organ support in pediatric patients with MOD and IAHS resulting from dengue shock syndrome (DSS).

**Methods:**

This retrospective observational research includes MOD and IAHS resulting from DSS. All children had 4 h of hemoperfusion with hemoadsorption for a duration of 1–3 consecutive days, accompanied by multimodal therapy. Inflammatory markers and mortality rates were assessed.

**Results:**

Five patients were included. All patients received hemoperfusion with hemoadsorption combined with CRRT, while four out of five patients received IVIG, dexamethasone, and plasmapheresis. The median PELOD‐2 and vasoactive‐inotropic score (VIS) decreased postintervention (8.0 vs. 6.0 and 20.0 vs. 10.0). The median log_10_ serum ferritin (5.000 vs. 4.724 ng/mL, *p* value < 0.05) and log_10_ serum IL‐6 (3.193 vs. 2.018 pg/mL, *p* value < 0.05) levels of the biomarker were significantly decreased. No additional adverse effects were noted.

**Conclusion:**

The combination of hemoperfusion with hemoadsorption and multimodal therapy effectively reduced inflammatory biomarkers and enhanced hemodynamic stability. Nevertheless, mortality outcomes should be assessed across larger populations.

**Highlights**

• The early use of hemoperfusion with hemoadsorption can reduce inflammatory cytokines in pediatric patients with MOD and IAHS due to DSS. This improves hemodynamic stability and might prevent eventual organ dysfunction.

## 1. Introduction

Dengue infection is a vector‐borne disease caused by *Aedes aegypti* that is most common in tropical and subtropical countries. The incidence of dengue varies by nation. In Thailand, the range is from 26.7 to 100.9 per 100,000 population for dengue hemorrhagic fever (DHF) and from 0.86 to 3.58 per 100,000 population for dengue shock syndrome (DSS) [1]. Most dengue infections occur in young children between the ages of 2 and 5 who live in urban areas [2]. The disease’s clinical course can go from an asymptomatic stage to a severe one with a high risk of morbidity and mortality. Multiple organ dysfunction (MOD) such as acute kidney injury (AKI), acute liver failure (ALF), myocardial dysfunction, and IAHS are atypical manifestations of dengue infection. ALF occurs in about 0.31%–18.5% of dengue cases, mostly in young adults [3, 4], and hemophagocytic syndrome occurs in about < 1% of dengue cases [5].

Both conditions including ALF and infection‐associated hemophagocytic syndrome (IAHS) can result in a hyperinflammatory state, which can induce hemodynamic instability and further MOD, particularly in hemophagocytic syndrome. Among the several pathophysiologies of IAHS, one is an increase in inflammatory cytokines and immunological dysregulations [6]. According to our understanding, removing inflammatory cytokines early can reduce systemic inflammation and prevent further organ damage. Multimodal treatment including intravenous immunoglobulin (IVIG), corticosteroid, therapeutic plasma exchange (TPE), hemoperfusion with hemoadsorption, and/or extracorporeal organ support can be used for stabilizing the patient and managing this condition. There are different types of cartridges, the hemoperfusion cartridge HA330 (Jafron Biomedical Co., Ltd., China) is a nonselective cytokine removal [7], HA330 being one of the most commonly used for cytokine removal in critically ill patients in intensive care units and a successful adjunctive treatment for cytokine removal in children with refractory septic shock [8].

We describe five cases of MOD and IAHS resulting from DSS. All of them had a hyperinflammatory state, and their clinical condition was deteriorating. Hemoperfusion with hemoadsorption and multimodal treatment including IVIG, corticosteroid, TPE, and/or extracorporeal organ support was used.

## 2. Materials and Methods

### 2.1. Ethics Statement

Protocol No. 0150/67 was approved by the Institutional Review Board of King Chulalongkorn Memorial Hospital, Faculty of Medicine, Chulalongkorn University, for the experimental protocols in this retrospective observational study. All methodologies employed in this investigation were executed in compliance with relevant regulations. All experiments were conducted following the Declaration of Helsinki, and all medical record evaluations and data collection were conducted under the hospital director’s authorization to access medical records, which waived the requirement for signed informed consent.

### 2.2. Study Design and Study Population

The researcher reviewed the medical records of individuals aged 1 month to 18 years diagnosed with DSS with IAHS and admitted to King Chulalongkorn Memorial Hospital from July 1, 2023, to October 31, 2023. The research included five patients. We reviewed the patient’s baseline characteristics, severity score, organ dysfunction score, duration of extracorporeal organ support, and first laboratory findings, which included a complete blood count, blood chemistry, liver function tests, coagulogram, and inflammatory biomarkers. All patients received early hemoperfusion with hemoadsorption and continuous renal replacement therapy (CRRT) within 24 h of admission, for 4 h per session across 1–3 consecutive days. Subsequent to hemoperfusion with hemoadsorption, the researcher performed daily TPE for ALF support. The first TPE session was performed within 24–36 h of admission. Four of the five patients received IVIG and dexamethasone for IAHS. Following therapy, laboratory results and clinical scores were obtained 24 and 48 h postinitial hemoperfusion with hemoadsorption and TPE, and results were analyzed.

### 2.3. Statistical Analysis

Each patient’s baseline characteristics, severity scores, organ dysfunction score, days of extracorporeal organ support, and laboratory results were described as numerical data. The function log_10_ was applied to the inflammatory biomarkers before analysis, and the clinical score and the function log_10_ of inflammatory biomarkers were reported as the median and interquartile range (IQR). The Wilcoxon signed‐rank test was used for statistical analysis. All data were analyzed with IBM SPSS Statistics software Version 25.0 (IBM Corp., NY, USA). A *p* value < 0.05 was considered statistically significant.

### 2.4. Study Definitions

#### 2.4.1. DSS

DSS is characterized as Grade 3 or Grade 4 clinical DHF with organ dysfunction of two or more systems according to Pediatric Organ Dysfunction Information Update Mandate (PODIUM), Contemporary Organ Dysfunction Criteria [9]. DHF Grade 3 includes narrow pulse pressure < 20 mmHg, weak pulse, prolonged capillary refill time > 2 s, or cold extremities, while Grade 4 includes profound shock with nondetectable pulse or blood pressure.

#### 2.4.2. IAHS

IAHS is an uncommon condition precipitated by viral or bacterial infections leading to secondary hemophagocytic lymphohistiocytosis (HLH) [10]. The disease’s pathophysiology consists primarily of dysregulation of natural killer T‐cell activity, leading to uncontrolled production of inflammatory cytokines, which results in MOD and a high mortality rate. The clinical manifestations consist of persistent high‐grade fever, splenomegaly, pancytopenia, a rise in inflammatory biomarkers, and MOD [5]. The patient who meets the criteria for diagnosing HLH‐2004 [11], fulfilling at least 4 criteria together with hemophagocytic activity in the bone marrow and presenting evidence of dengue viral infection, was diagnosed with IAHS. The patient diagnosed with IAHS was treated with dexamethasone 10 mg/m^2^/day and an intravenous infusion of IVIG 1 g/kg over 10–12 h.

#### 2.4.3. CRRT

CRRT is a technique to remove body substances and fluids, which is suitable for patients who are hemodynamically unstable in the intensive care unit due to low fluid removal rates [12, 13]. We use CRRT on all patients, with the Prismaflex System (Baxter; Gambro Industries, Meyzieu, France) and M100 filter. In all patients, the dialysis catheter was inserted into the right internal jugular vein; for patients who initiated VA–extracorporeal membrane oxygenation (ECMO), we connected the CRRT circuit to the VA‐ECMO circuit. All patients were started on CRRT with continuous venovenous hemodiafiltration (CVVHDF), and the CRRT dose ranged from 2 to 4 L/1.73 m^2^/h. Due to an abnormal liver function test, the regional citrate anticoagulant (RCA) was administered at a lower dose than usual, 1.5–2.0 mmol/L of citrate in the filter. The blood chemistry and ionized calcium (iCa) were monitored for signs of citrate intoxication. In patients on VA‐ECMO, we used systemic heparin instead of RCA, with a starting dosage of 10 units/kg/h.

#### 2.4.4. TPE

TPE is a technique for removing water‐soluble and protein‐bound toxins in patients with ALF [14]. We use a blood cell separator machine from the blood bank to separate plasma and replace it with fresh frozen plasma (FFP). All patients received high‐volume TPE for at least 3 consecutive days, which was 1.5–2.0 times the volume of their plasma. The plasma volume was calculated using the formula: total body water × (1‐hematocrit) [15]. All patients received intravenous 10% calcium gluconate to prevent hypocalcemia during TPE, and no patient had a serious allergic reaction to a blood product during TPE.

### 2.5. Hemoperfusion With Hemoadsorption Techniques

Hemoperfusion with hemoadsorption is a procedure that uses extracorporeal organ support for removing unwanted inflammatory cytokines [16]. This approach removes inflammatory cytokines using adsorption from sorbent polymer in a cartridge connected to extracorporeal organ support. There are various types of sorbent polymers depending on the range of material removal [17]. In this study, we used HA330‐II (Jafron Biomedical Co., Ltd.), whose sorbent polymer is polystyrene divinylbenzene. This cartridge is for broad‐spectrum inflammatory cytokine removal. We connected it to the CRRT machine and utilized it for 4 h each session.

## 3. Results

### 3.1. Patient Characteristics and Treatment Modalities

The study included five pediatric patients diagnosed with severe DSS and IAHS, aged 10–14 years old. The data are presented in Table 1. The earliest fever on admission was fever on Day 2, followed by fever on Day 6, which was a late presentation. The severity score at admission (pretreatment) was evaluated for all patients, including the vasoactive‐inotropic score (VIS), which were 10, 35, 75, 20, and 7 in Patients 1‐5, and the Pediatric Logistic Organ Dysfunction 2 (PELOD‐2) score, which were 7, 13, 14, 8, and 5 in Patients 1–5. Patient number 2 had a maximum Pediatric Index of Mortality 3 (PIM‐3) score of 55.5% at admission. All pediatric patients received early hemoperfusion with hemoadsorption and high‐dose CRRT in CVVHDF mode. Patient numbers 1–4 received PLEX, while all patients except Patient number 3 received IVIG and dexamethasone. Two patients received ECMO, and all of them were intubated and connected to a mechanical ventilator. After early hemoperfusion with hemoadsorption and multimodal treatment, the severity score, including VIS and PELOD‐2, was evaluated in all patients, as shown in Table 1.

**Table 1:** Patients’ characteristics and treatment modalities.

Case	Age (years)	Days of fever on admission	VIS (pretreatment)	VIS (posttreatment)	PIM‐3 (%)	PELOD‐2 Pretreatment	PELOD‐2 Posttreatment	Hemoperfusion cartridge	Hemoperfusion (days of fever)	Multimodal treatment	ECMO days	CRRT days	Ventilator days
1	12	5	10	5	28.6	7	3	HA330‐II	d6, d8	IVIG, Dex, TPE	—	6	9
2	10	6	35	15	55.5	13	7	HA330‐II	d7, d8	IVIG, Dex, TPE	—	6	11
3	14	2	75	25	32.4	14	12	HA330‐II	d3, d4	TPE	8	8	9
4	11	6	20	10	17.0	8	6	HA330‐II	d7, d9, d11	IVIG, Dex, TPE	4	6	6
5	10	5	7	0	15.7	5	5	HA330‐II	d6	IVIG, Dex	—	3	3

*Note:* PELOD‐2 = Pediatric Logistic Organ Dysfunction 2 score; IVIG = intravenous immunoglobulin; Dex = dexamethasone; ECMO = extracorporeal membrane oxygenation.

Abbreviations: CRRT = continuous renal replacement therapy; OI = oxygenation index; PIM‐3 = Pediatric Index of Mortality 3; TPE = therapeutic plasma exchange; VIS = vasoactive‐inotropic score.

### 3.2. Organ Dysfunction and Laboratory Parameters

Table 2 shows organ dysfunction characteristics for all patients, according to the PODIUM criteria. The highest organ dysfunction was 8 organ dysfunction systems, which occurred in Patient numbers 2 and 3, while others had 7 organ dysfunction systems. The laboratory parameters in all pediatric patients included hemoglobin, hematocrit, absolute neutrophil count (ANC), platelet count, serum lactate, prothrombin time (PT), international normalized ratio (INR), aspartate aminotransferase (AST), alanine aminotransferase (ALT), gamma glutamyl transferase (GGT), total bilirubin, direct bilirubin, and serum ammonia. These parameters are shown in Table 3 and were evaluated at admission (pretreatment), as well as after posttreatment^∗^ and posttreatment^∗∗^. Laboratory posttreatment^∗^ was performed after the first session of hemoperfusion with hemoadsorption following the first TPE session. Laboratory posttreatment^∗∗^ was performed after the second session of hemoperfusion with hemoadsorption following the second TPE session in Patients 2 and 3 and after the third TPE session in Patients 1 and 4.

**Table 2:** Organ dysfunction characteristics in patients with dengue shock syndrome.

Case	Neurologic	Respiratory	Cardiovascular	Renal	Gastrointestinal	Hepatic	Hematology	Coagulation	Endocrine	Immune
1	Yes	Yes	Yes	Yes	No	Yes	Yes	Yes	No	No
2	Yes	Yes	Yes	Yes	No	Yes	Yes	Yes	Yes	No
3	Yes	Yes	Yes	Yes	No	Yes	Yes	Yes	Yes	No
4	Yes	Yes	Yes	Yes	No	Yes	Yes	Yes	No	No
5	Yes	Yes	Yes	Yes	No	Yes	Yes	Yes	No	No

*Note:* Organ dysfunction is reported according to the PODIUM criteria.

**Table 3:** Comparison of laboratory parameters between pre‐ and posttreatment.

Blood parameter	Case no. 1			Case no. 2			Case no. 3			Case no. 4			Case no. 5	
	Pretreatment	Posttreatment^∗^	Posttreatment^∗∗^	Pretreatment	Posttreatment^∗^	Posttreatment^∗∗^	Pretreatment	Posttreatment^∗^	Posttreatment^∗∗^	Pretreatment	Posttreatment^∗^	Posttreatment^∗∗^	Pretreatment	Posttreatment^∗^
Hemoglobin (g/dL)	12.7	11.6	12.4	9.7	12.6	11.3	10.9	8.1	7.9	8.4	7.0	7.9	13.5	9.1
Hematocrit (%)	38.9	34.2	35.5	28.8	37.2	32.7	34.4	24.1	21.2	27.3	23.0	24.0	39.7	26.6
ANC (× 10^3^/μL)	4.6	4.1	6.6	8.9	10.3	10.3	7.2	20.2	19.3	17.9	16.9	18.1	14.66	5.65
Platelet (× 10^3^/μL)	19.0	17.0	39.0	50.0	52.0	74.0	109.0	95.0	104.0	53.0	73.0	94.0	37.0	100.0
Serum lactate (mg/dL)	10.5	5.9	4.3	16.2	3.9	3.5	17.4	6.0	8.6	8.9	15.6	7.8	1.5	3.3
PT (sec.)	23.6	16.2	14.6	22.1	17.2	15.7	41.6	39.0	26.8	34.7	60.2	28.6	19.3	14.9
INR	2.1	1.4	1.2	2.0	1.5	1.4	4.0	3.7	2.4	3.1	5.5	2.5	1.7	1.3
AST (U/L)	19,262	5472	2912	15,476	5322	2266	1707	3591	2531	18,964	11,384	7673	10,496	2286
ALT (U/L)	2748	639	483	1762	781	545	575	1477	1697	3473	1909	1393	3306	1642
GGT (U/L)	606	191	175	167	107	—	—	—	—	71	—	—	186	—
Total bilirubin (mg/dL)	3.9	3.2	3.1	2.7	6.5	5.9	0.87	1.71	3.24	5.51	5.12	10.94	1.45	1.12
Direct bilirubin (mg/dL)	3.0	2.2	1.8	1.8	4.5	4.2	0.42	0.91	1.99	1.53	0.83	1.66	1.0	0.77
Ammonia (μmol/L)	66.2	62.0	44.6	286.5	134.6	111.3	120.6	98.0	91.6	347.4	330.0	255.2	127.6	70.1

*Note:* AST = aspartate aminotransferase; ALT = alanine aminotransferase.

Abbreviations: ANC = absolute neutrophil count; GGT = gamma‐glutamyl transferase; INR = international normalized ratio; PT = prothrombin time.

^∗^After the 1^st^ session of hemoperfusion with hemoadsorption.

^∗∗^After the 2^nd^ session of hemoperfusion with hemoadsorption.

### 3.3. Inflammatory Biomarkers and Clinical Scores

All patients had inflammatory biomarkers and clinical scores evaluated before and after hemoperfusion with hemoadsorption treatment. Inflammatory biomarkers included serum ferritin, interleukin‐6 (IL‐6), and high‐sensitivity C‐reactive protein (hsCRP). Figure 1 shows that all inflammatory biomarkers showed a decreasing tendency in all patients between pretreatment and posttreatment. We used the log_10_ algorithm before analyzing the data and presented the results as median with IQR as shown in Table 4. Log_10_ ferritin and log_10_ IL‐6 levels significantly differed between pretreatment and posttreatment, with medians of 5.000 and 4.724 (*p* value < 0.05) and 3.193 and 2.018 (*p* value < 0.05), respectively. The median log_10_ hsCRP decreased between pretreatment and posttreatment, but not statistically significantly; the values were 1.632 and 1.522 (*p* value > 0.05), respectively. We compared clinical scores, including VIS and PELOD‐2, pretreatment and posttreatment. Table 4 shows a significant difference in clinical scores between pretreatment and posttreatment: VIS was 20.000 and 10.000 (*p* value < 0.05), and PELOD‐2 was 8.000 and 6.000 (*p* value < 0.05).

Figure 1Alterations in the blood chemistry profile of five patients before and after treatment. Before treatment = pre, after 1^st^ session of hemoperfusion with hemoadsorption = post‐1, after 2^nd^ session of hemoperfusion with hemoadsorption = post‐2. The data from each patient are shown as log_10_ transformation: (a) log_10_ IL‐6, (b) log_10_ ferritin, and (c) log_10_ hsCRP.

**Figure:**
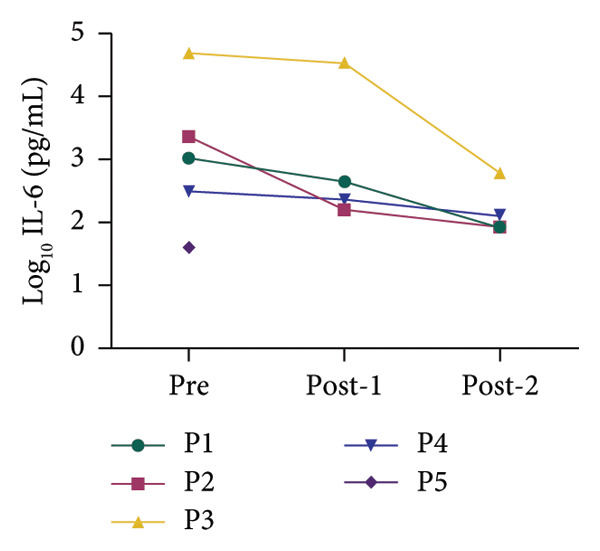
(a)

**Figure:**
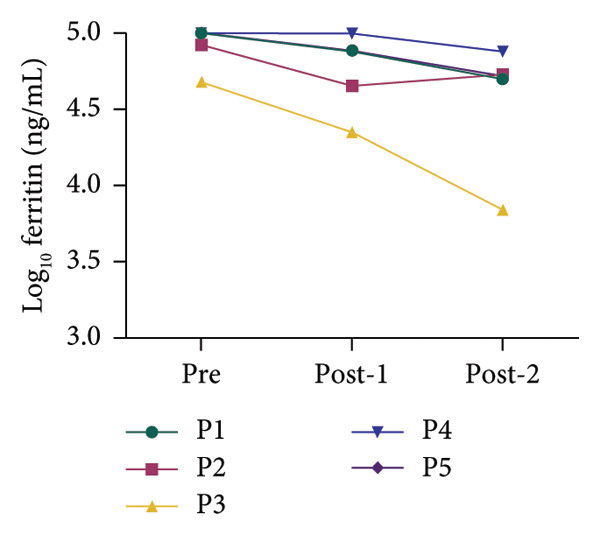
(b)

**Figure:**
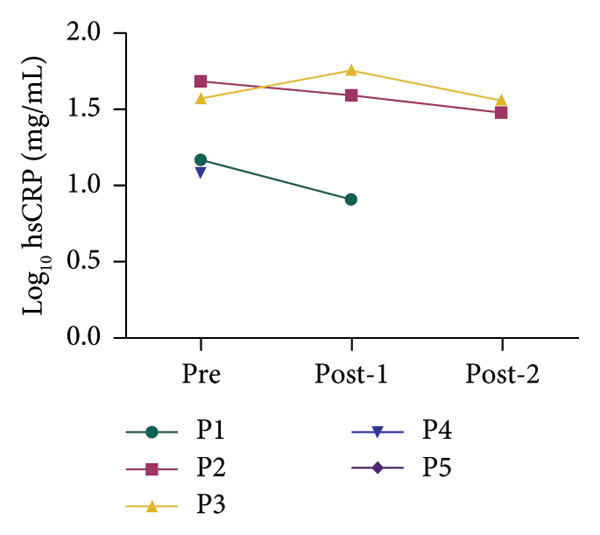
(c)

**Table 4:** Inflammatory biomarkers and clinical scores before and after treatment with hemoperfusion.

Parameters	Pretreatment	Posttreatment	*p* value
Log_10_ ferritin (ng/mL)	5.000 (0.196)	4.724 (0.534)	0.027
Log_10_ IL‐6 (pg/mL)	3.193 (1.744)	2.018 (0.694)	0.016
Log_10_ hsCRP (mg/mL)	1.632 (0.108)	1.522 (0.086)	0.231
VIS	20.000 (46.500)	10.000 (17.500)	0.046
PELOD‐2	8.000 (7.500)	6.000 (5.500)	0.026

*Note:* IL‐6 = interleukin‐6; PELOD‐2 = Pediatric Logistic Organ Dysfunction 2 score. The data are presented as median with interquartile range (IQR).

Abbreviations: hsCRP = high‐sensitivity C‐reactive protein; VIS = vasoactive‐inotropic score.

## 4. Discussion

This study aims to evaluate the effectiveness of hemoperfusion with hemoadsorption and multimodal treatment for multiple organ failure and hemophagocytic syndrome in pediatric patients suffering from severe DSS. The liver is one of the organs most often affected by dengue virus infection, potentially resulting in ALF. The pathophysiology of ALF in dengue viral infection may be attributed to the dengue virus itself or immunological mechanisms [18, 19]. ALF can result in an accumulation of toxic substances and the production of excessive inflammatory cytokines, leading to MOD, with a mortality rate of around 68.3% [20]. Another condition that may be triggered by dengue virus infection is IAHS. HLH can be divided into primary HLH and secondary HLH. IAHS is a form of secondary HLH, mostly induced by infections, mostly viral in origin [21]. Dengue‐associated HLH is an unusual condition that requires early recognition in cases of dengue infection presenting with atypical features and a hyperinflammatory state, because the condition has been linked to increased mortality rates [22, 23].

In children with severe DSS, the immune system’s inability to cope with the viral infection and both of these conditions can result in excessive inflammation, known as a cytokine storm, leading to MOD and fatality. Most extracorporeal liver support systems use TPE to eliminate inflammatory cytokines and toxins while waiting for liver recovery. The primary treatment for IAHS is immune‐modulating medication, including IVIG and dexamethasone. Some reports indicate that the combination of TPE with immune‐modulating treatment enhanced survival outcomes in IAHS [24, 25]; however, this is unsatisfactory, maybe resulting from excessive inflammation and subsequent organ damage. According to our knowledge, early use of hemoperfusion with hemoadsorption can reduce inflammatory cytokines, reduce the clinical effects of cytokine storms, and enhance organ function, which makes it an effective adjunctive treatment for children with IAHS and MOD, including ALF resulting from dengue viral infection, thereby improving survival rates [26].

There are insufficient data on the use of hemoperfusion with hemoadsorption in IAHS for children with severe DSS; nonetheless, several reviews and recommendations encourage its application in critically ill patients during the COVID‐19 pandemic [27]. Some studies indicate efficacy in reducing inflammatory cytokines and enhancing organ function in ALF [28] and septic shock [29, 30]. Our study shows an improvement in VIS and PELOD‐2 scores following the early use of hemoperfusion with hemoadsorption within 24 h of admission, when combined with immune‐modulating medication and extracorporeal organ support in the majority of children. We observed a significant reduction in inflammatory biomarkers, including serum ferritin and IL‐6, both of which are the most important inflammatory biomarkers in HLH and cytokine storms. The HA330‐II is a broad‐spectrum cytokine removal therapy; our study used it as an adjunctive treatment, showing a correlation between improved organ function and a reduction in inflammatory biomarkers without significant adverse events. This study has several limitations, including a small sample size due to the rarity of the illness. We did not present the mortality outcome. We lost half of them during hospitalization owing to fungal infection and irreversible brain injury, despite improvements in organ function and a decreasing trend in inflammatory biomarkers. We did not assess the effects of therapy between the use of immune‐modulating medication with extracorporeal organ support alone and hemoperfusion with hemoadsorption. The outcome following treatment may be more closely associated with the overall therapeutic approach than with hemoperfusion and hemoadsorption specifically. Further research is required for future comparisons between patients who use hemoperfusion with hemoadsorption and those who do not.

## 5. Conclusion

Early hemoperfusion with hemoadsorption within 24 h of admission combined with multimodal treatment including IVIG, dexamethasone, TPE, and/or extracorporeal organ support was effective in decreasing inflammatory biomarkers, improving hemodynamic stability, and eventually restoring vital organ functions in pediatric patients with MOD and IAHS from severe DSS. However, mortality rates should be compared across larger populations. The effect of hemoperfusion with hemoadsorption alone versus standard treatment alone should be compared.

## Ethics Statement

This retrospective observational study received approval for all experimental protocols from the Institutional Review Board of King Chulalongkorn Memorial Hospital, Faculty of Medicine, Chulalongkorn University (Protocol No. 0150/67).

## Consent

The authors have nothing to report.

## Conflicts of Interest

The authors declare no conflicts of interest.

## Author Contributions

Tananat Virojtriratana was responsible for conceptualization, methodology, formal analysis, data curation, visualization, and original manuscript writing.

Kanruetai Na Nan was responsible for conceptualization and data curation.

Rujipat Samransamruajkit was responsible for conceptualization, methodology, and supervision of the project.

## Funding

No funding was received for this study.

## Data Availability

The datasets used and/or analyzed during the current study are available from the corresponding author on reasonable request.
